# To branch or to expand?

**DOI:** 10.7554/eLife.17079

**Published:** 2016-06-06

**Authors:** Esther M Bridges, Adrian L Harris

**Affiliations:** Molecular Oncology Laboratories, Oxford University Department of Oncology, Weatherall Institute of Molecular Medicine, John Radcliffe Hospital, Oxford, United Kingdom; Molecular Oncology Laboratories, Oxford University Department of Oncology, Weatherall Institute of Molecular Medicine, John Radcliffe Hospital, Oxford, United Kingdomadrian.harris@oncology.ox.ac.uk

**Keywords:** angiogenesis, agent based modelling, neovascularization, signalling dynamics, Human, Mouse

## Abstract

Oscillating protein signals control the branching and expansion of blood vessels.

**Related research article** Ubezio B, Blanco RA, Geudens I, Stanchi F, Mathivet T, Jones ML, Ragab A, Bentley K, Gerhardt H. 2016. Synchronization of endothelial Dll4-Notch dynamics switch blood vessels from branching to expansion. *eLife* **5**:e12167. doi: 10.7554/eLife.12167

**Image** High levels of the protein VEGF increase the diameter of blood vessels

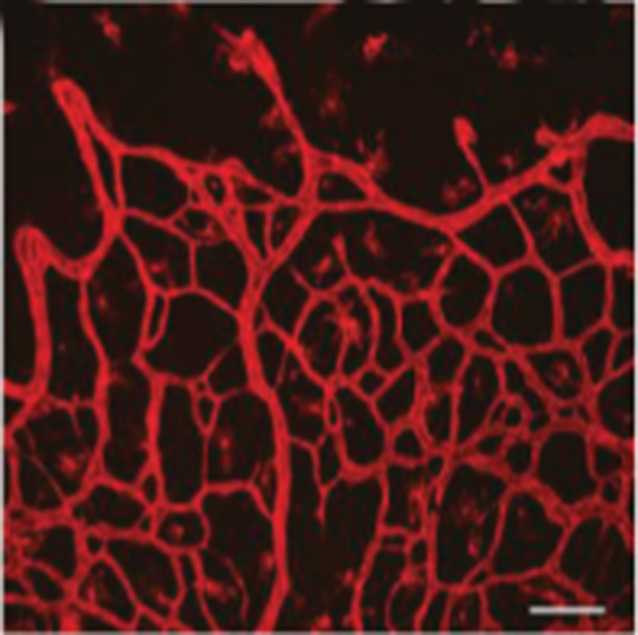

The network of blood vessels in the body needs to be continuously repaired and extended to enable it to supply sufficient oxygen and nutrients to tissues. New blood vessels are created from existing ones in a complex process called angiogenesis. Normally, this process is tightly controlled because it can also promote the progression of many diseases, such as inflammatory arthritis and cancer. However, little is known about the mechanisms that determine whether a vessel should create a new branch, or widen existing vessels (a process that is known as expansion).

A well established model for the control of blood vessel branching involves a feedback loop between two key signal proteins called vascular endothelial growth factor A (VEGF) and Delta-like four (Dll4) (Blanco and Gerhardt, 2013). Tumour cells or pro-inflammatory cells that are starved of oxygen release VEGF, which activates the branching process by binding to a receptor on so-called tip cells (Figure 1A). These cells are a type of endothelial cell found on the inner surface of blood vessels. They have outgrowths called filopodia that sense the VEGF signals and respond by moving towards the source.

**Figure 1.**
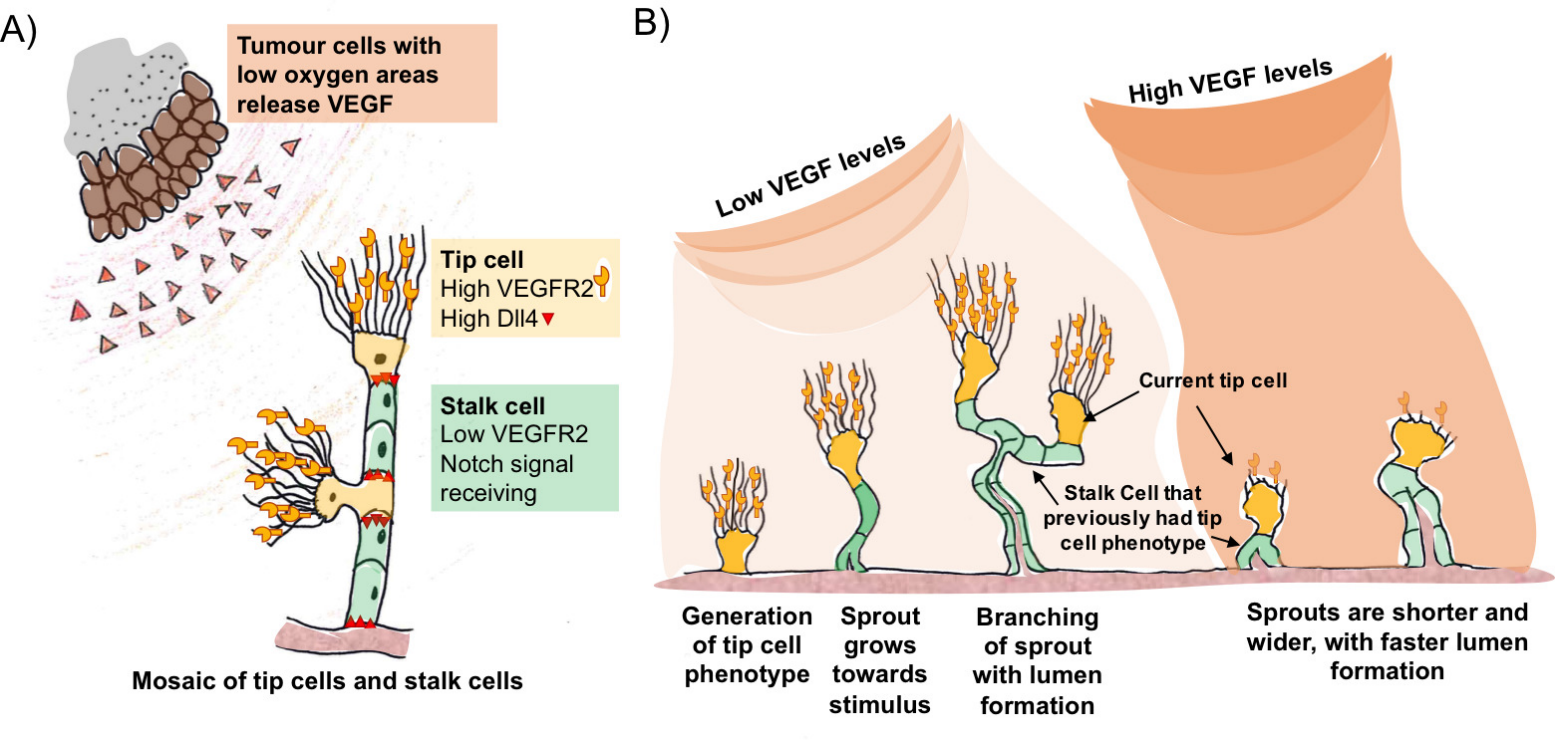
VEGF and Dll4 regulate blood vessel branching and expansion. (**A**) Tumour cells in areas of low oxygen levels release VEGF, which is detected by receptors (VEGFR2) on tip cells (yellow). This triggers the tip cells to display more Dll4 proteins on their surface. Dll4 binds to Notch receptors on neighbouring cells (green), which leads to them becoming stalk cells. This results in a new branch of the vessel (sprout) forming with a mosaic pattern of tip and stalk cells, where the tip cells are separated by stalk cells. (**B**) The mosaic pattern of tip and stalk cells is not permanent and changes as the new sprout grows towards the source of the VEGF signal. Ubezio, Blanco et al. used fluorescent reporters to show that some stalk cells have previously been tip cells. The levels of VEGF increase as the sprout expands and high levels of VEGF (which also lead to high levels of Dll4) disrupt the mosaic pattern and lead to stunted sprouting, reduced branching and allow the vessel to expand.

VEGF triggers an increase in the amount of Dll4 protein on the surface of the tip cells. Dll4 can bind to a receptor protein called Notch that is found on neighbouring endothelial cells. This reduces the amount of VEGF receptor on these so-called stalk cells, preventing them from responding to VEGF signals. This process – referred to as Dll4/Notch lateral inhibition – generates an evenly scattered “mosaic” arrangement of tip cells that are separated by no more than two stalk cells (Bentley et al., 2014; Claxton and Fruttiger, 2004).

The mosaic pattern is not a permanent feature. As the tip cells move out from the existing blood vessel, the stalk cells follow to create the new branch (Figure 1B). The stalk cells then multiply so that the tip cells are now separated by larger numbers of stalk cells, which enables the new branch of the vessel (the sprout) to grow towards the source of the signal. VEGF and Dll4/Notch lateral inhibition are responsible for the continuous competition between these cell types as the sprout grows towards the VEGF gradient. Now, in eLife, Katie Bentley and Holger Gerhardt at the London Research Institute and colleagues – including Benedetta Ubezio and Raquel Blanco as co-first authors – report that Dll4 levels fluctuate in individual endothelial cells as the sprouts form (Ubezio et al., 2016).

Ubezio, Blanco et al. – who are based at the London Research Institute and Vesalius Research Centre – combined video microscopy data with computer simulations to predict and demonstrate how a network of blood vessels could develop. By monitoring the levels of Dll4 messenger ribonucleic acid (mRNA) in cultured single layers of endothelial cells, they found that the production of DII4 in these cells oscillated in response to VEGF. In cells exposed to low levels of VEGF, Dll4 mRNA fluctuated up and down in 4-5 hour cycles. In contrast, high levels of VEGF resulted in a much larger peak of Dll4 mRNA at about 8 hours that was dependent on Notch signalling. In more complex models, high VEGF led to the formation of shorter vessels with wider diameters. The vessels contained tip cells arranged in clusters instead of an alternating mosaic pattern.

Cell culture experiments using fluorescent reporters that respond to Dll4 confirmed that the mosaic pattern of tip and stalk cells seen in animals could be reproduced under low levels of VEGF. Next, Ubezio, Blanco et al. manipulated the developing blood vessels within the eyes of newborn mice by artificially increasing VEGF levels, and blocking the multiplication of stalk cells. This enabled them to exclude cell division as an explanation for the change in vessel size. This confirmed that high VEGF levels synchronise the Dll4 signal across many cells and drive vessel expansion. Furthermore, the fluorescent reporters show that some of the stalk cells in the expanding vessels have previously been tip cells. In a mouse model of glioblastoma – a type of brain tumour – vessel branching was associated with a scattered pattern of Dll4 expression, indicating that the tip and stalk cells were arranged in a mosaic pattern. On the other hand, increasing the levels of Dll4 or VEGF in the eyes disrupted vessel branching and the scattered Dll4 pattern, and promoted vessel expansion.

Why is it important to understand how blood vessels decide to branch or expand in tumours? Drugs that inhibit angiogenesis have been developed to treat cancer and although they slow the progression of the disease, they rarely increase the lifespan of the patient (Bridges and Harris, 2011). The loss of filopodia from VEGF-induced sprouts due to high VEGF levels and the synchronisation of Dll4 signals may lead to vessels expanding in tumours. This could be an aspect of vascular normalisation, where blood vessel walls become less permeable to blood plasma proteins and the flow of blood along the vessel increases (Wong et al., 2015; Jain, 2013). However, many tumours do not show this effect and the enlarged vessels that remain may still be detrimental to the patient because of the increased delivery of oxygen to the tumour and the clearance of waste molecules (Li et al., 2011).

Some studies report that inhibiting VEGF signalling can improve the delivery of drugs that block the growth of tumours (Batchelor et al., 2013), while other studies report the opposite effect (Pham et al., 2015). By understanding how Dll4/Notch lateral inhibition and VEGF signalling regulate angiogenesis, new therapies can be developed to target essential phases of blood vessel growth.

Many questions about angiogenesis remain. For example, how do the tip and stalk cells move towards the source of the VEGF signal? What are the mechanisms that control their ability to adhere to each other (Dejana and Lampugnani, 2014)? How are other types of cells recruited to the tip and stalk cells to form the outside wall of the vessel, and what effect does nutrient starvation have on angiogenesis (Bridges and Harris, 2011; Pham et al., 2015)? With the model developed by Ubezio, Blanco et al., we can expect a much deeper understanding in the near future.
